# Notes and Notices

**Published:** 1893-10

**Authors:** 


### NOTES AND NOTICES.

Dr. John Storer removed September 1st, 1893, to corner Revere and Alveston Streets, Jamaica Plain, Mass.

Dr. A. W. Holcombe has located at Kokomo, Indiana, in the office with Dr. Sawyer. He has the honor of being the first graduate from the Hering College of Homœopathy.

The Purity of Cocaine has been investigated lately, and the results of it may be found in the statement on advertising page 5.

---

### FUN FOR DOCTORS.

In a Hospital.—Doctor (to patient)—" Young man, you do not seem to pick up as fast as I expected you would."

Patient—" That's so, doctor; I don't feel as if I would be able to leave the hospital for some time yet. I believe that the nurse is to blame for it."

" Why, how is that?"

" Well, she is only eighteen years old, and very good looking."

" I think I'll have to prescribe another nurse."—*Texas Siftings.*

Husband—" Dr. Foote, the chiropodist, will dine with us this evening."

Wife—"All right; I'll order corned beef."

Losing Faith in Editors.—Old Lady—" I don't believe this Sure Cure Tonic is a-goin' to do me any good."

Friend—" It's highly spoken of in the papers."

Old Lady—" Yes, but I've taken forty-seven bottles, and I don't feel a bit better. I tell you what it is, Sarah, I'm beginning to think these newspaper editors don't know everything."—*New York Weekly.*

